# Using Single *loxP* Sites to Enhance Homologous Recombination: ts Mutants in *Sec1* of *Dictyostelium discoideum*

**DOI:** 10.1371/journal.pone.0000724

**Published:** 2007-08-08

**Authors:** Mark S. Bretscher, Margaret Clotworthy

**Affiliations:** Medical Research Council Laboratory of Molecular Biology, Cambridge, United Kingdom; Massachusetts Institute of Technology, United States of America

## Abstract

**Background:**

*Dictyostelium discoideum* amoebae are haploid and, as they share many features with animal cells, should be an ideal creature for studying basic processes such as cell locomotion. Isolation of mutants in this amoeba has largely been limited to non-essential genes: *nsfA*—the gene for NEM-sensitive factor—remains the only essential gene for which conditional (ts) mutants exist. These ts mutants were generated by gene replacement using a library of mutagenised *nsfA* containing a selectable marker: transformants were then screened for temperature sensitivity. The success of this approach depended on the high level of homologous recombination prevailing at this locus: ∼95% of selected clones were homologous recombinants. This is unusually high for *Dictyostelium*: homologous recombination at other loci is usually much less, usually between 0–30%, making the isolation of ts mutants much more tedious.

**Methodology/Principal Findings:**

In trying to make ts mutants in *sec1A*, homologous recombination was found to be only ∼25%. A new approach, involving single *loxP* sites, was investigated. *LoxP* sites are 34 bp sequences recognised by Cre recombinase and between which this enzyme catalyses recombination. A *Dictyostelium* line containing a single *loxP* site adjacent to the 3′ end of the *sec1A* gene was engineered. A *sec1A* replacement DNA also containing a single *loxP* site in a homologous position was then introduced into this cell line. In the presence of CRE recombinase, homologous recombination increased to ∼80% at this locus, presumably largely driven by intermolecular recombination between the two single *loxP* sites.

**Conclusions/Significance:**

A route to increase the rate of homologous recombination at a specific locus, *sec1A*, is described which enabled the isolation of 30 ts mutants in *sec1A*. One of these, *sec1Ats1*,has been studied and found to cease moving at the restrictive temperature. The approach described here may be valuable for enhancing homologous recombination at specified loci and thus for introducing mutations into specific genes in *Dictyostelium* and other creatures.

## Introduction

With a haploid genome and known genomic sequence, the *D. discoideum* amoeba is perhaps the most attractive organism for studying the motile machinery and how this is integrated with signalling processes in chemotaxis. Methods have been available for some time for removing non-essential genes in *Dictyostelium* and many of these, particularly those associated with the cytoskeleton, have been inactivated [1]. However, the most effective way of analysing the functions of essential genes is through temperature sensitive (ts) mutants and, to date, such mutants have been isolated in only one gene, *nsfA*, a gene whose function is required for many steps in intracellular membrane transport. At the restrictive temperature, the phenotype of *nsfA* showed that it is unsurprisingly required for several membrane processes, such as fluid phase endocytosis and phagoctyosis. Interestingly, however, NSF is also required for cell locomotion [2], supporting the view for a dynamic role for membranes in movement. The isolation of these ts mutants depended both on the earlier demonstration that homologous recombination can occur between an introduced replacement vector and the genome [3], [4] and on the unusually high level of homologous recombination which was found at this site: ∼95% of isolated transformants were found to be homologous replacements [2]. This enabled mutagenised replacement DNAs to substitute the native gene and a screen to be performed to find ts mutants. Other genes are more refractory to this approach; this is largely due to the lower level of homologous recombination which prevails at these other loci. This makes it more difficult to screen a mutagenised library and, in turn, to isolate temperature sensitive mutants. The aim of the present work was to obtain ts mutants in the single-copy gene, *sec1A*. Sec1 is a member of the Sec1/Munc18 family of proteins which, in combination with SNARE pairs, control membrane fusion [5]–[7]. Sec1 itself is required for exocytosis in yeast and, as there is only one gene homologous to Sec1 in *Dictyostelium*, it seemed likely that it too would be essential and required for exocytosis. As membrane circulation may be of prime importance for cell movement [8], [9] it seemed of particular interest to discover what effect the loss of sec1p activity would have.

**Figure 1:**
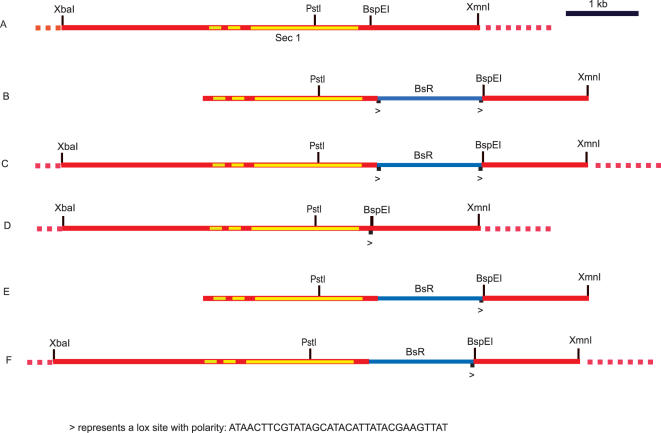
The *sec1A* locus of *Dictyostelium* and constructs. (A) Layout of genomic site of *sec1A*; in each figure the dots at each end of a DNA stretch indicate that that DNA is part of the genome. Sec1p coding sequence in yellow, the floxed blasticidin cassette (BsR) in blue, the rest of the genome in red. (B) Transforming DNA used to measure homologous recombination at this site. (C) Layout of the gene after homologous recombination has occurred between the genome in A and the DNA in B. (D) Transforming the *Dictyostelium* line, shown in C, with Cre recombinase leads to the loss of the blasticidin cassette, yielding the *sec1A* strain SD5, having a single inserted 3′ *loxP* site. (E) Singly loxed transforming DNA used to measure homologous recombination and carry out mutagenesis. (F) Layout of the *sec1A* gene after homologous recombination between strain SD5 and the DNA in E.

In preliminary experiments we found that the rate of homologous recombination at the *sec1A* locus (Fig 1A) is ∼25%; when a replacement vector (Fig 1B) bearing the selectable marker—a blasticidin cassette—just beyond the C-terminus of the gene was introduced into amoebae, only 25% of the blasticidin-resistant clones were homologous recombinants. In the remaining 75% the cassette had integrated elsewhere in the genome. A new route was devised which raised the level of homologous recombination to ∼80% and this has enabled several useful ts mutants to be isolated. The same procedure may assist in the isolation of ts mutants in other genes in *Dictyostelium* and perhaps can be adapted for use in other organisms.

The underlying idea behind this procedure takes advantage of recent advances in which *Dictyostelium* genes can be deleted successively using a selectable marker—the floxed blasticidin S cassette [10]. The cassette, flanked by two *loxP* sites, is introduced into a target gene by homologous recombination. The cassette is then removed from the genome by Cre recombinase in an intramolecular circularisation event which removes the blasticidin cassette so that the cell line is once again blasticidin-sensitive. This allows other genes to be disrupted in succession. When excision occurs, a single *loxP* site remains in the target. For the present method, the relevant point is that it is possible to introduce single *loxP* sites into the genome in this round-about fashion. This poses the question: if a gene replacement vector has only a single *loxP* site, and if the target gene itself has a single appropriately placed *loxP* site, would site-specific recombination, by an intermolecular recombination event, be enhanced in the presence of Cre recombinase?

## Results and Discussion

The layout of the *sec1A* region is shown in Fig 1A. A standard vector, with a blasticidin cassette about 200bp downstream from the 3′ end of the gene, was constructed (Fig 1B). When introduced into Ax2 amoebae, the rate of homologous recombination was found to be ∼25% (see Table 1). In other experiments (not shown) two vectors carrying a new restriction marker site, *NgOM*IV, (designed not to alter the sec1p sequence and placed ∼600bp before the PstI site) were constructed, one having an in-phase UAA codon next to the NgOMIV site and the other without. Homologous recombinants bearing the NgOMIV site could not be obtained with the first vector (see Table 1), whilst many were obtained with the second. In other words, no recombinants having an in-phase termination codon could be isolated. This indicates that *sec1A* is an essential gene, a line of evidence previously used for *nsfA* [2]. This in turn implied that it should be possible to isolate ts mutants of *sec1A*.

**Table 1:** Levels of Homologous recombination using different replacement DNAs.

	Strain	
	Ax2	SD5
Replacement DNA	Homologous Recombination	
No *loxP* sites (as in Fig 1B)	25% (7/30)	
No *loxP* sites (but with internal stop codon)	0% (0/30)	
Single *loxP* site (as in Fig 1E)	25% (13/50)	80% (32/39)
NSF gene 1	95%	

1 Taken from [2]

To try to improve the rate of homologous recombination as outlined in the Introduction, a cell line transformed with the vector (Fig 1B) was isolated (Fig 1C). These cells were then transfected with a vector expressing Cre recombinase and, using G418 selection, a blasticidin-sensitive clone isolated (Fig 1D). This strain, SD5, having a single *loxP* site at the 3′ end of *sec1A* and which retained its G418-sensitivity (and hence expression of Cre recombinase) was used for the rest of the experiments reported here.

A new replacement vector was constructed, identical to that in Fig 1B but having a single *loxP* site (with the same orientation as in Fig 1B) at the 3′ end of the blasticidin cassette (Fig 1E). When linearised and introduced into SD5, the rate of homologous recombination was measured: ∼80% of the clones were homologous recombinants (Table 1; producing the strain shown in Fig 1F). As a control, when the same replacement DNA was introduced into Ax2, the rate of homologous recombination was, as expected, ∼25% (see Table 1). This shows that homologous recombination can be substantially enhanced if homologous single *loxP* sites are included in both the vector and the host and in the presence of Cre recombinase.

**Figure 2:**
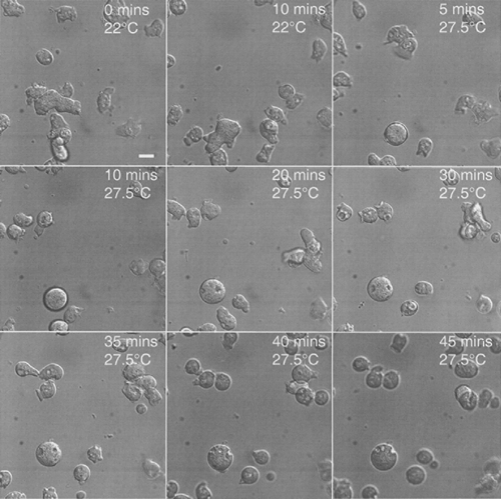
Locomotory behaviour of *sec1A1*. Cells were plated out in growth medium in a small optical chamber and allowed to attach to the glass underslip. The cells, examined using a microscope fitted with a heated stage, are shown at 22°C and after an interval of 10 mins: the temperature was then shifted to 27.5°C and the cells followed for a further 45 mins. *sec1A1* cells move normally at 22°C, but progressively lose this ability at 27.5°C, so that after about 30 mins, most have a rounded appearance and cease translocating. They do, however, continue to put out small protrusions. Scale bar: 10μ.

**Figure 3:**
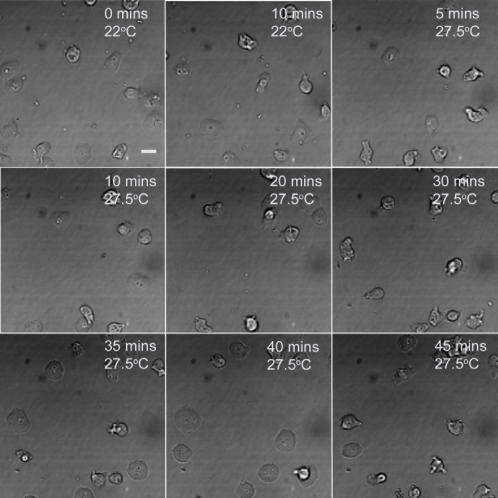
Locomotory behaviour of the parental line, *Ax2.* Cells were plated out as in Fig 2. The cells are shown at 22°C for 10 mins: the temperature was then shifted to 27.5°C and the cells followed for a further 45 mins. *Ax2* cells move normally at 22°C and continue to do so at 27.5°C. Scale bar: 10μ.

This procedure was used to isolate ts mutants in *sec1A* using a heavily mutagenised library, as described previously for *nsfA* [2]. Some 1240 SD5 transformed clones were screened, yielding 30 independent ts mutants in sec1p, showing incidentally that *sec1A* is an essential gene in *D. discoideum*. One of these mutants (*sec1A1* or HM1163) has been reconstructed and studied in some detail [11]. In particular, these cells cease to migrate at the restrictive temperature (Fig 2) whereas the parental line, Ax2, continues freely to do so (Fig 3). Although the cells largely stop translocating, they do change their shape and put out protrusions. This defect in motility recovers after the cells are returned to the permissive temperature. The motile behaviour of this sec1A mutant is very similar to that seen in ts mutants of NSF at the restrictive temperature, where the cells′ appearance was likened to a “rabbit in a sack” [2].

There are many situations in which an enhanced rate of homologous recombination would be valuable for gene replacements. Preliminary experiments suggest that the orientation of the *loxP* site with respect to the gene is important: *loxP* sequences are not symmetrical. It could be that a very high rate of homologous recombination could be engineered at a given locus by placing two *loxP* sites, in opposite orientations, at either end of the locus and using a replacement vector having both *loxP* sites in homologous positions.

## Methods

### Cloning: *Sec1A* vectors with 1–2 *loxP* sites

The genomic sequence of *sec1A* (DDB 0231656), including ∼1500 bp 3′ sequence, was assembled in BlueScript: different parts were either isolated by genomic cloning or by PCR. That these pieces had the correct sequence was determined by sequencing. A unique marker *NgOMIV* site, which is helpful in distinguishing the replaced gene from the original vector, was introduced by PCR at ∼860 bp into the genomic sequence of *sec1A* (converting ATT.*GCA.GGT.*TTT to ATT.*GCC.GGC.*TTT and thus not changing the amino acid sequence). A unique *BspEI* site, located ∼200 bp beyond the end of the coding sequence, was used to introduce the selective cassette: this contained either one *loxP* site (at that end of the cassette remote from the 3′ end of *sec1A*, near the 3′ end of the blasticidin gene) or two *loxP* sites (one at each end of the cassette, [10]).

### Transformation and clonal analysis

Ax2 amoebae (or derivatives of Ax2) were transformed by electroporation with linearised DNA according to the procedure of Knecht [12]. After electroporation, 4×10^6^ cells were diluted into axenic medium, dispersed into 3 96-well plates and grown for 1 day before addition of blasticidin S (to a final concentration of 10 µg/ml). They were grown for a further 10–14 days: about 30–50 of the larger clones were then picked and recloned onto bacterial plates. DNA was prepared from each clone (with a Sigma mammalian DNA isolation kit, G-1N 70).

Homologous recombinants were identified by pcr with the primers: TCAGATTTCTCTGCAGAGAAGT and GTTGATAACCGGAGGGTCTTG, which amplify the C-terminal region of the *Sec1A* gene from the PstI site to just beyond the original BspEI site. Ax2 yields a ∼0.8 kb band, a homologous recombinant only a ∼2.2 kb band. Likewise, when a blasticidin-resistant amoeboid line was treated with Cre recombinase, it became blasticidin sensitive and PCR showed that the blasticidin cassette had been removed. For isolating ts mutants, a mutagenic library (with a complexity of about 50,000 and having 1–4 bases mutated/100 bp) was used to generate ts mutants.
